# Supplementary material for: From healthy to unhealthy obesity: A longitudinal study of adults in ELSA-Brasil
Source: PLOS Glob Public Health. 2025 Apr 29;5(4):e0004325. doi: 10.1371/journal.pgph.0004325 (PMC12040175; doi:10.1371/journal.pgph.0004325)
Supplement: Attachment — Submitted filename: PLOS Global Public Health_OBMS.pdf [file pgph.0004325.s001.pdf]

Introduction
In the second paragraph of the introduction, the current wording suggests that the authors already assert the nonexistence of metabolically healthy obesity (MHO). I recommend revising this section to provide a balanced discussion of the evidence both supporting and opposing this condition (lines 48-62, page 7).

Additionally, the phrasing of the hypothesis implies that the group assumes MHO to be a transitional state. Given the current limitations in knowledge on this topic, I suggest rethinking how this hypothesis is presented to better reflect the uncertainties and nuances still under investigation.

Methods
What is the reason for not using data from the second wave to track weight variation over the years?

Overall, the methods are well-described and meet the requirements of the proposed analyses, but including this information could enhance the understanding of the approach adopted.

Results
I would like to understand why the variables associated with changes in body weight over the years were not presented. Were these data analyzed? If so, did they have a relationship with MHO?

I believe this analysis would be crucial to better understand the relationship between obesity diagnosed by BMI and the observed metabolic changes.

Discussion
In line 325, I suggest paying attention to the use of acronyms and recommend replacing them with full terms for better clarity.

Overall, the discussion is well-grounded and incorporates important references on the topic. However, it would be valuable to include reflections on the challenges of caring for people with obesity and the experience of living with a chronic and recurrent condition. It is urgent to construct new narratives that help move beyond practices focused exclusively on weight loss and the pathologization of larger bodies.

I invite the authors to reflect on the importance of care strategies that do not reinforce blame and, instead, promote the development of more empathetic and inclusive practices that support the maintenance of a healthy life regardless of weight loss. Metabolically healthy obesity is a complex issue that can contribute to the development of broader and health-centered indicators rather than relying solely on weight or the exclusive use of BMI as a monitoring parameter.

Conclusion Revision
The evidence found highlights alcohol consumption and increased waist circumference as risk factors for metabolic transition. This underscores the need to promote healthy habits as central components of obesity care.

Considering obesity as a chronic and recurrent condition, we emphasize that care must go beyond an exclusive focus on weight loss. It is essential to incorporate balanced eating, regular physical activity, moderation in alcohol consumption, stress management, and other aspects. These measures can contribute to preventing metabolic changes and promoting overall well-being.
