# Supplementary material for: From healthy to unhealthy obesity: A longitudinal study of adults in ELSA-Brasil
Source: PLOS Glob Public Health. 2025 Apr 29;5(4):e0004325. doi: 10.1371/journal.pgph.0004325 (PMC12040175; doi:10.1371/journal.pgph.0004325)
Supplement: Attachment — Submitted filename: RESPONSE TO THE REVIEWERS - MHO - PLoS ONE 04022025.docx [file pgph.0004325.s003.docx]

**Dears Editor and Reviewers:**

We want to thank you for your attention and comments on the manuscript and for allowing us to submit a revised draft of our paper. We have made all the adjustments indicated by the reviewers. All changes are marked in yellow. Here is a point-by-point response to the reviewers’ comments and concerns. We improved the text according to the suggestions made by all the reviewers, and we hope that this new version presents better quality.

**Comments from the Editors and Reviewers:**

**Reviewer #1:**

1. MHO as a transitory state, which could be revised to reflect the limitations of current knowledge on the subject. Regarding the discussion, I recommend including reflections on the challenges of caring for individuals with obesity, highlighting the importance of overcoming practices focused exclusively on weight loss and the pathologization of larger bodies. Considering obesity as a chronic and recurrent condition, it is crucial to emphasize empathetic and comprehensive care strategies that promote healthy lifestyles and embrace individual experiences. These adjustments could significantly contribute to enhancing the relevance and applicability of the results, both in the clinical context and in the development of more inclusive health policies.

**Response:** Thank you for your consideration. In the last paragraph of the conclusion (Lines 378 - 387), reflections were included on the care of patients with obesity, as well as the issue of self-esteem and the importance of promoting healthy habits, rather than focusing solely on weight loss.

Introduction

2. In the second paragraph of the introduction, the current wording suggests that the authors already assert the nonexistence of metabolically healthy obesity (MHO). I recommend revising this section to provide a balanced discussion of the evidence both supporting and opposing this condition (lines 48-62, page 7).

**Response:** Thank you for your consideration. The section has been rewritten to reinforce the duality of scientific knowledge on the topic (lines 52–58).

3. Additionally, the phrasing of the hypothesis implies that the group assumes MHO to be a transitional state. Given the current limitations in knowledge on this topic, I suggest rethinking how this hypothesis is presented to better reflect the uncertainties and nuances still under investigation.

**Response:** Thank you for your consideration. Lines 57-59. The hypothesis has been rewritten, emphasizing that it is testing whether MHO is indeed a transitional state or not.

Methods

4. What is the reason for not using data from the second wave to track weight variation over the years?

**Response:** It was decided to use only wave 1 and wave 3 of the study to ensure a longer follow-up period for the participants, allowing for a more accurate and detailed analysis of changes in metabolic status over time.

Results

5. I would like to understand why the variables associated with changes in body weight over the years were not presented. Were these data analyzed? If so, did they have a relationship with MHO? I believe this analysis would be crucial to better understand the relationship between obesity diagnosed by BMI and the observed metabolic changes.

**Response:** To assess changes in body weight over the years between baseline and wave 3, BMI and waist circumference were used. Body weight alone was not considered, as it is already included in the calculation of BMI and, moreover, it can vary significantly based on height, making its isolated analysis less informative.

Discussion

6. In line 325, I suggest paying attention to the use of acronyms and recommend replacing them with full terms for better clarity.

**Response:** Thank you for your consideration. The terms were replaced for greater clarity for the readers.

7. Overall, the discussion is well-grounded and incorporates important references on the topic. However, it would be valuable to include reflections on the challenges of caring for people with obesity and the experience of living with a chronic and recurrent condition. It is urgent to construct new narratives that help move beyond practices focused exclusively on weight loss and the pathologization of larger bodies. I invite the authors to reflect on the importance of care strategies that do not reinforce blame and, instead, promote the development of more empathetic and inclusive practices that support the maintenance of a healthy life regardless of weight loss. Metabolically healthy obesity is a complex issue that can contribute to the development of broader and health-centered indicators rather than relying solely on weight or the exclusive use of BMI as a monitoring parameter.

**Response:** Thank you for your consideration. Thank you for your consideration. In the last paragraph of the conclusion (Lines 378 - 387), reflections were included on the care of patients with obesity, as well as the issue of self-esteem and the importance of promoting healthy habits, rather than focusing solely on weight loss.

Conclusion Revision

8. The evidence found highlights alcohol consumption and increased waist circumference as risk factors for metabolic transition. This underscores the need to promote healthy habits as central components of obesity care. Considering obesity as a chronic and recurrent condition, we emphasize that care must go beyond an exclusive focus on weight loss. It is essential to incorporate balanced eating, regular physical activity, moderation in alcohol consumption, stress management, and other aspects. These measures can contribute to preventing metabolic changes and promoting overall well-being.

**Response:** Thank you for your consideration. The above comment was taken into consideration, and changes were made in the discussion and conclusion to reflect the points raised in the suggestion.

**Reviewer #2**

Methods

1. Line 112: "Food intake was analyzed longitudinally (dynamic model),...".The authors need to better explain or reference the model used to estimate food intake
   **Response:** Thank you for your consideration. A more detailed explanation of how the variable was calculated has been provided, and it can be found on lines 124 - 127.
2. Line 116 - 119: "The International Physical Activity Questionnaire (IPAQ) long version, validated for Brazil by Matsudo et al. (23), was applied. Physical activity was recorded in minutes per week and subdivided into low, moderate, and high categories, according to the World Health Organization (24)."It is not clear how the physical activity variable was calculated and categorized. What are the cutoff points? It is necessary to justify the choice of the term low, moderate, and high.The studies, for the most part, classify whether the individual is inactive, insufficiently active or active.
   **Response:** Thank you for your consideration. The information has been added to the text. The measurement of physical activity in minutes per week was made by multiplying the weekly frequency by the duration of each activity performed. The categorization proposed by the WHO (2010) was used, which defines insufficient, moderate, and vigorous physical activity, recommending at least 150 minutes of moderate physical activity per week or 75 minutes or more of vigorous physical activity. Thus, the cut-off points were established based on the WHO guidelines to classify the intensity of physical activity as low, moderate, or high.

Results

3. Age was divided into quartiles. Report the cutoff points for each category
   **Response:** Thank you for your consideration. The age range for each time point was added to the tables.
4. In the tables, inform in the legend what O1 and O3 of the variables mean: % of grams of MPF O1 and O3, % of grams of PF O1 and O3 and % of grams of UPF O1 and O3
   **Response:** Thank you for your consideration. The abbreviations were changed and replaced with W1 and W3, which respectively indicate "Wave 1" and "Wave 3."
5. Line 244-246: "Also, the final regression was tested using the follow up duration as a potential confounder and it yielded extremely similar results, demonstration no significant effect on the risk factors identified or their measures of association."
   The authors need to explain why they considered follow-up time as a confounding factor. By considering follow-up time in the analysis, the measure of occurrence and association would change.
   **Response:** The follow-up time of the participants was considered a confounding factor in the study due to the variation in the timing of data collection for each participant, which took place over a period of one and a half years. This difference in the timing of data collection can contribute to a significant difference in the follow-up time of individuals, which may influence metabolic changes and the progression of health indicators. By adjusting the analyses for follow-up time, we ensure a more accurate assessment of the associations between the variables of interest and minimize the influence of temporal discrepancies on the results.

Discussion

6. It would be easier for the reader if the authors, when comparing with the literature, informed the study population and location of the cited reference. For example:

Line 261-262: "The work of Palatini and colleagues indicated that the MHO state is an unstable condition, as after 7.5 years of follow up, 59.3% of MHO individuals transitioned to an unhealthy status (5)."

**Response:** The change has been made, lines 274 - 275.

Line 286-287: "Among these patients, 86.9% were individuals with 287 obesity, 27.7% determined as having diabetes, and 78.2% had elevated fasting glucose levels."

**Response:** The change has been made, lines 298 - 300.

Line 319-322: "Another study conducted a 30-year follow-up to observe the 320 transition of metabolic status and stated that women move in and out of the MHO classification over the years, while men do not; after transitioning from MHO to MUO status, they generally remain in the unhealthy state(6)."

**Response:** The change has been made, line 333.

7. **REGARDING JOURNAL REQUIREMENTS**
1. Please provide separate figure files in .tif or .eps format.

**Response:** Figures are provided as requested.

2. All PLOS journals now require all data underlying the findings described in their manuscript to be freely available to other researchers, either
   a. In a public repository, 
   b. Within the manuscript itself, or 
   c. Uploaded as supplementary information.

**Response:** The ELSA-Brasil (Brazilian Longitudinal Study of Adult Health) operates under a well-defined protocol governing the use of its database. The study maintains a closed data policy to ensure the confidentiality and proper management of participant information. Access to the database is strictly regulated and can only be granted upon reasonable request. Researchers interested in utilizing ELSA-Brasil data must submit a formal application detailing the objectives and scope of their proposed study. All requests for data access and publication are subject to review by a dedicated Publication Committee. This committee carefully assesses each proposal to ensure compliance with ethical and scientific guidelines, safeguarding the integrity of the database and the privacy of participants.

Other studies using the ELSA-Brasil dataset have been published by PLOS and usually the information on data availability is presented as follows:

Due to ethical restrictions approved by the ethics committee of each institution (Universidade Federal de Minas Gerais, Universidade de São Paulo, Universidade Federal do Espírito Santo, Universidade Federal do Rio Grande do Sul, Universidade Federal da Bahia e Fundação Oswaldo Cruz) and by the Publications Committee of ELSA-Brasil (publiELSA), the data used in this study can be made available for research proposals by a request to ELSA's Datacenter ([rb.sgrfu@asleacitsitatse](mailto:rb.sgrfu@asleacitsitatse)) and to the ELSA's Publications Committee. Additional information can be obtained from the ELSA Coordinator from the Research Center of Espírito Santo ([jose.mill@gmail.com](mailto:jose.mill@gmail.com)).

This paragraph has been added to the manuscript.

3. Please review your reference list to ensure that it is complete and correct. If you have cited papers that have been retracted, please include the rationale for doing so in the manuscript text, or remove these references and replace them with relevant current references. Any changes to the reference list should be mentioned in the rebuttal letter that accompanies your revised manuscript. If you need to cite a retracted article, indicate the article’s retracted status in the References list and also include a citation and full reference for the retraction notice.

**Response:** All references have been reviewed.
